# The Effectiveness of Autologous Platelet Concentrates in the Clinical and Radiographic Healing after Endodontic Surgery: A Systematic Review

**DOI:** 10.3390/ma16227187

**Published:** 2023-11-16

**Authors:** Alessandro Espedito di Lauro, Alessandra Valletta, Angelo Aliberti, Mario Cangiano, Pasquale Dolce, Gilberto Sammartino, Roberta Gasparro

**Affiliations:** 1 Department of Neurosciences, Reproductive Sciences and Odontostomatological Sciences, University of Naples Federico II, 80131 Naples, Italy; alessandroespedito.dilauro@unina.it (A.E.d.L.); alessandra.valletta@unina.it (A.V.); ange.aliberti@studenti.unina.it (A.A.); mario.cangiano3@studenti.unina.it (M.C.); roberta.gasparro@unina.it (R.G.); 2 Department of Public Health, University of Naples Federico II, 80131 Naples, Italy; pasquale.dolce84@gmail.com

**Keywords:** endodontic surgery, platelet-rich fibrin, autologous platelet concentrates, postoperative pain, apicoectomy

## Abstract

Regenerative techniques are increasingly applied in endodontic surgery, but different materials may have varying impacts on soft and hard tissue healing. This systematic review aims to evaluate the effectiveness of autologous platelet concentrates (APCs) in clinical and radiographic healing after endodontic surgery. The data for this systematic review were processed following the PRISMA (Preferred Reporting Items for Systematic Reviews and Meta-Analyses) guidelines for improving the reporting of systematic reviews and meta-analyses. A literature search was conducted until October 2023 on PubMed, Scopus, and Cochrane Databases. Randomized controlled trials and controlled clinical trials addressing the use of APCs in patients who presented persistent periapical lesions and needed periapical surgery were included. Dual publications, narrative reviews, systematic reviews, case series, questionnaires, animal studies, case reports, letters to the editor, in vitro studies, and abstracts were excluded. In total, the search resulted in 14 papers. Clinical and radiographical findings were reported, showing that when APCs were used, patients exhibited less pain and swelling and a greater reduction of apical radiolucency after 12 months follow-up on average. However, the moderate/high risk of bias of included studies and their high heterogeneity, do not allow one to draw definitive conclusions on the effectiveness of APC after endodontic surgery.

## 1. Introduction

The necrosis of pulp tissue not adequately treated can lead to periapical periodontitis, which is the complex of inflammatory pathologies of the periapical tissues of the tooth (alveolar bone and periodontal ligament) [1,2]. Endodontic surgery consists of the reduction or elimination of persistent periapical pathology when primary orthograde endodontics or retreatment have failed or are not feasible [3]. Historically, conventional endodontic surgery, which involved larger surgical access and less sophisticated instrumentation, often presented challenges in achieving predictable outcomes. Nevertheless, with the advent of microsurgical techniques and the integration of cutting-edge technologies, the field of endodontics has evolved significantly [4]. However, considering that endodontic surgery is linked to a less predictable prognosis compared to orthograde endodontic treatment [5] and even a single tooth can be strategic in the whole oral prosthetic rehabilitation, the possibility of accelerating bone regeneration in periapical surgical defects could be of great interest to the clinician to proceed earlier with permanent rehabilitation.

The following several methods have been used to promote bone regeneration and soft tissue healing in periapical defects as an adjunct to endodontic surgery: barrier membranes, bone grafting materials, bone morphogenetic proteins (BMPs), platelet-derived growth factor (PDGF), and enamel matrix proteins (EMD) [6]. In particular, non-resorbable expanded polytetrafluoroethylene (e-PTFE) and bioabsorbable collagen have been commonly used as they can prevent the apical migration of epithelial cells and facilitate the repopulation of the bony defect by osteogenic cells; furthermore, bone grafts can preserve the necessary space for new bone formation, supply essential osteogenic cells to promote bone growth (osteogenic effect), stimulate host cells to regenerate lost bone tissue (osteoinductive effect), and act as supportive frameworks (osteoconductive effect). Some reviews have supported the use of regenerative techniques in endodontic surgery [7,8], but others have reached negative conclusions [9,10]. So, the effectiveness of their application is still questionable and remains a subject of ongoing investigation and debate.

In recent years, autologous platelet concentrates (APCs) have been introduced as an autologous grafting material in several different fields of dentistry [11,12]. 

APCs are autologous blood products used in several medical and dental fields to increase soft and hard tissue healing rate [13]. 

Several technical procedures have been developed to obtain different platelet concentrates with variable yield of platelets and cellular components [14].

APCs can be classified into platelet-rich plasma (PRP) and platelet-rich fibrin (PRF) based on distinct preparation processes. PRP, as the first-generation platelet concentrate, is plasma with a high platelet concentration obtained through specific centrifugation of fresh whole blood. PRF, on the other hand, as the second-generation platelet concentrate, is characterized by strong fibrin polymerization obtained during the centrifugation process. This procedure requires blood collection without an anticoagulant and immediate centrifugation for the formation of a fibrin clot, which includes not only platelets but also leukocytes [15,16].

APCs can be thought of as a reservoir of growth factors as platelet-derived growth factor (PDGF), transforming growth factor beta (TGF-β), etc., which have been involved in cell proliferation, chemotaxis, and extracellular matrix production/angiogenesis [17,18], fibroblast growth factors (FGF) 1 and 2 and vascular endothelial growth factor (VEGF) which play critical roles in the hemostasis, proliferative, and remodeling phases of wound healing [19,20]. Platelet degranulation also leads to the release of cytokine and chemokines, such as interleukin (IL)-1β, C-C motif ligand 5 (CCL5), IL-8, and macro- phage inflammatory protein (MIP)-1α, which contribute to the healing process [21]. Moreover, the use of APCs as an adjunct in oral surgery was reported to add beneficial effects in terms of pain relief and an improvement of postoperative quality of life [22].

The application of APCs in endodontic surgery has already been described in recent clinical cases and in a randomized clinical trial in the specific field of treatment of apicomarginal defects [23]. However, their application in this field is still questionable and the benefits they provide to both surgeon and patient have been reported to be moderate and remain controversial. Moreover, few systematic reviews, including all types of APCs and different methods of analysis, were published in the literature. 

Thus, the aim of this systematic review was to evaluate the effectiveness of APCs in terms of clinical outcomes and radiographic healing in patients undergoing apical surgery and evaluate whether the design of the primary studies may affect the results. The null hypotheses in most of the articles included in the above review predicted that periapical surgical defects filled with APCs would require the same healing time as sites treated with conventional surgical techniques and that patients would experience the same postoperative discomfort with or without application of the APC.

## 2. Methods

### 2.1. Focused Question

The data for this systematic review were processed following PRISMA (Preferred Reporting Items for Systematic Reviews and Meta-Analyses) guidelines [24]. According to the PICO criteria (P: population, I: intervention, C: comparison, O: outcome) statement, this review aimed to answer the following question: “Do autologous platelet concentrates provide benefits in terms of reduced postoperative discomfort and pain (Clinical Outcomes) and accelerated radiographic healing (Radiographic Outcomes) in patients (Population) undergoing endodontic procedures (Intervention)?” Physiologic healing of the surgical site provided by the blood clot or biomaterials was used as a comparison/control. The protocol was registered in the International Prospective Register of Systematic Reviews (PROSPERO) with number CRD42023401240.

### 2.2. Search Strategy 

To prepare the study protocol, a pilot search was performed on the PubMed search platform, followed by a systematic evaluation of potentially suitable studies for inclusion in the study. At the end of the pilot search, data extraction forms were drafted. The literature search was conducted by consulting three electronic databases (PubMed, Scopus, and The Cochrane Library) until October 2023, using keyword combinations and MeSH terms, according to the database rules (Table 1).

The manual search also included a search from the following journals: Giornale Italiano di Endodonzia, Journal of Endodontics, Journal of Clinical Periodontology, International Journal of Periodontics and Restorative Dentistry, Clinical Oral Investigation, Clinical Oral Implant Research, International Surgery, Implant Dentistry, Quintessence International, Journal of Prosthodontic, International Journal of Prosthodontics, European Journal of Oral Implantology, Journal of Oral and Maxillofacial Surgery. In addition, an attempt was made to search the grey literature by searching for potentially suitable studies among conference abstracts published on WoS and Scopus and scientific dental conference databases. Two authors (AA, RG) searched the articles independently and resolved disagreements by discussing their search results.

### 2.3. Inclusion and Exclusion Criteria 

Studies were selected based on the following inclusion criteria:

1. The study was randomized controlled trials (RCTs) or clinical controlled trials (CCTs);
2. Patients presented with persistent periapical lesions and needed periapical surgery;
3. APCs were utilized in the intervention group(s);
4. Physiologic healing or regenerative materials alone or combination of APCs and regenerative materials instead of APC were utilized in the control group;
5. Reported clinical or radiographical outcomes or both.

Studies were excluded based on the following exclusion criteria:

1. Dual publications, narrative reviews, systematic reviews, case series, questionnaires, animal studies, case reports, letters to the editor, in vitro studies, abstracts;
2. Outcomes of interest were not extractable;
3. Articles written in any language other than English;
4. Full text not available.

### 2.4. Selecting and Extracting Data from Studies

**Figure 1:**
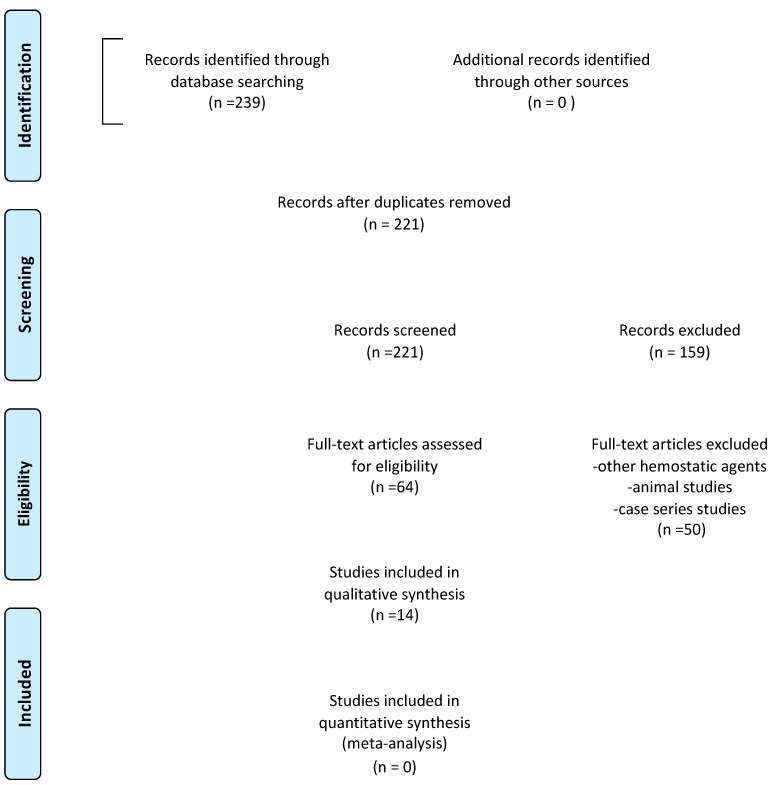
Prisma flow diagram.

The selection process is reported in Figure 1 (PRISMA flow diagram). The titles and abstracts, when available, of all articles identified through electronic searches were independently analyzed by two authors (AA, RG). For studies that appeared to meet the inclusion criteria, or for those for which there was insufficient data in the title and abstract, the entire article was consulted. Full articles obtained from all search methods, electronic and otherwise, were independently evaluated by two authors (AA, RG) to determine whether the studies met the inclusion criteria. Discrepancies were resolved by discussion, and if resolution was not possible, a third review author was consulted (AV). The main reasons for the exclusions of title, abstract, and full text were the following: study design (case report/case series), animal studies, regenerative technique, hemostatic agents instead of APCs, and full text not available. All studies that met the inclusion criteria were then subjected to risk-of-bias assessment and data extraction. Data were extracted by two review authors independently (AV, RG), using specially drafted data extraction forms, and any discrepancies were resolved with discussion. The following data were recorded for each of the following articles, as shown in Table 2 (characteristics of the included studies): author of the study, year of publication and country in which the research was carried out, type of study, sample size, age and sex of the patients involved in the study, diagnosis, type of intervention performed, control group analyzed in each study (when present), follow-up, diagnostic methodology, and clinical and radiographic results. 

### 2.5. Methodological Quality of Included Reviews

Two authors (AA and RG) independently assessed the studies in terms of inclusion criteria, relevance, eligibility, and risk of bias following the recommendations of the Joanna Briggs Institute Critical Appraisal tool (JBI) [39]. Any disagreement was solved by consensus between reviewers and statisticians (PD). The JBI does not provide a range of scores that indicate the overall quality but considering the relative importance of each domain and its potential impact on the study results and interpreting the domain scores in the context of the study, the studies were classified as having a low risk of bias if most domains score “Yes”, a moderate risk if some domains are rated “No/Unclear”, and a high risk if multiple domains have significant bias or are rated “No”.

## 3. Results

### 3.1. Search Results

From the initial search, 239 studies were identified from electronic databases (PubMed, Scopus, and The Cochrane Library), while no studies were selected through other sources. After removing duplicates, the titles, and abstracts of 221 articles were analyzed. Of those selected, 159 articles were deemed unsuitable by title and abstract and were therefore excluded. The remaining 64 articles were deemed eligible according to the pre-established eligibility criteria. On examining the full text of these articles, case reports, case series, systematic reviews, and narrative reviews were excluded, and finally 14 articles were then included in the review. A total of 14 studies were included in this systematic review, including 9 randomized controlled trials [25,26,27,28,30,31,35,37,38] and 5 clinical controlled trials [29,32,33,34,36]. Some of them exclusively evaluated the clinical outcomes as postoperative discomfort, patient quality of life, infection, pain, postoperative swelling, and presence/absence of bleeding [27,30,35,36]. Others only analyzed radiographic healing of periapical bone tissue [25,31,32,33]. Only six studies evaluated both outcomes [26,28,29,34,37,38]. A meta-analysis was not possible due to the heterogeneity of statistical measures and outcomes used, and the poor statistical methodology quality of some studies.

### 3.2. Summary of Clinical Findings

In the study of Del Fabbro et al. [27], the addition of the liquid form and clot of PRGF after endodontic surgery gave a significant reduction in pain and swelling, fewer postoperative analgesics taken, and improved functional activities (mouth opening, chewing, speaking, sleeping, daily routines, and work) compared with patients in the control group. Similar results were obtained by Taschieri et al. [36] in terms of less pain and swelling when PRGF was used in situations of Schneiderian membrane perforation that occurred during endodontic surgery. In Soto-Peñaloza et al. [35], 50 apical lesions of the maxillary upper jaw were treated with and without A-PRF as an adjunctive treatment and showed that pain perception and quality of life (functional limitations and other symptoms) were significantly lower in the A-PRF test group. In contrast, Meschi et al. [30] showed no statistically significant difference (*p* ≤ 0.05) between the test and control groups in terms of VAS, occurrence of pain symptoms, impairment of daily activities, and medication use, daily in the 7 days following endodontic surgery, using L-PRF clot. In the study by Singh et al. [34], pain, mobility, swelling, and the vitality of adjacent teeth after apical endodontic surgery were evaluated between the following three groups: hydroxyapatite granules, CERAMENT bone, and PRF. PRF reduced pain, swelling, tooth mobility, and bleeding compared with hydroxyapatite and CERAMENT. Similarly, Angerame et al. [26] showed that patients in the PRF-treated group (test group) experienced less pain in the postoperative 2–6 h and developed less edema, which was always limited and intraoral. Finally, Dhiman et al. [28] and Goyal et al. [29] analyzed periodontal parameters, including pocket depth (PD), clinical attachment level (CAL), and gingival marginal position (GMP). Dhiman et al. [28] revealed that only pocket depth showed a statistically significant reduction in the test group treated with PRF. On the other hand, the study by Goyal et al. [29] found that PRP showed a similar reduction in periodontal pocket depth, clinical attachment level, and gingival margin position in comparison with collagen membrane and PRP + collagen sponge group. 

In Yahata et al. [38], regarding the VAS scores, there was no significant difference between the CGF and control groups preoperatively and at all postoperative appointments.

Finally, in Thakur et al. [37], regarding the parameters used for predicting quality of life, patients in the PRF Medium group reported significantly less swelling on the first, second, and third days postoperatively and less than average second, third, and fourth days postoperatively. There were significant differences in postoperative pain intensity (based on mean VAS scores) observed on the first, second, third, and fourth days postoperatively, with patients treated with PRF Medium showing consistently less pain.

### 3.3. Summary of Radiographic Findings

The radiographic healing of periapical bone tissue was detected by using different diagnostic methods. Meschi et al. [31], Ahmed et al. [25], and Parihk et al. [33], used cone beam computed tomography (CBCT). Specifically, Meschi et al. [31] found no improvement in bone healing when L-PRF was combined with a root surgical treatment compared to the same treatment without L-PRF; Parihk et al. [33] found that the PRP-treated site showed better healing as early as 8 weeks, and CBCT after 1 year showed an increase in periapical bone density in relation to the PRP-treated site. Ahmed et al. [25] found a significant volumetric reduction of a periapical lesion after 1 year as well when PRF was used alone. On the other hand, Monga et al. [32], Dhiman et al. [28], Goyal et al. [29], and Angerame et al. [26] performed evaluations of radiographic outcomes via a digital X-ray system using periapical radiographs. Specifically, Monga et al. [32] conducted a study of 30 patients with periapical radiolucency in maxillary anterior teeth. After 9 months, a significantly higher radiographic healing rate was observed in group PRF+MTA (82.36%). In Dhiman et al. [28], no significant differences were observed in the size of periapical lesions at 12-month follow-up between PRF group and spontaneous healing.

In Angerame et al. [26], periapical radiographs were taken before and after surgery and at each follow-up visit. The study showed that at recalls 2 and 3 months after surgery, the test group treated with PRF showed significantly better periapical radiographic healing scores than the control group. Thereafter, the periapical healing scores of the control and test groups were similar, and statistical analysis showed no significant differences.

In Yahata et al. [38], the total success rate assessed using periapical radiography at 12 months was 91.7%. Although no significant difference was observed in the success rates between the CGF and control groups evaluated by periapical radiography and CBCT, the lesion volume reduction rate was 75.6% in the CGF group and 61.0% in the control group, with a significantly higher reduction rate in the former. 

Finally, in Thakur et al. [37], significant improvements were seen in the size of the periapical lesion (SPL), and the volume of the periapical lesion (VPL) at the 12-month follow-up when compared with baseline in both groups. Specifically, buccal bone formation was observed in 26% of cases in the PRF Medium group and in 20% of cases in the PRF High group, with no significant difference between the groups. 

### 3.4. Risk of Bias Assessment 

The results of the bias risk assessment for randomized controlled trials (RCTs) and controlled clinical studies included in the review are shown in Table 3 and Table 4, respectively. For RCTs, five studies had a low risk of overall bias, three moderate, and only one high. The most perplexing domains were as follows: “Were participants blind to treatment assignment?” and “Were those delivering treatment blind to treatment assignment?” For CCTs, three had a moderate risk of overall bias and two high. 

## 4. Discussion

The present systematic review aimed to assess the effectiveness of APCs in clinical and radiographic healing after endodontic surgery. The findings revealed that when APCs were used, patients showed a significant reduction in postoperative pain and swelling and a greater reduction in apical radiolucency after 12 months of follow-up on average. In this review, it was observed that Del Fabbro, Soto-Penaloza, and Taschieri [27,35,36] reported more significant improvements in clinical outcome measures, including VAS (visual analogue scale) pain scores, functional outcomes, and QoL (quality of life) scores, which were not corroborated by the findings of the study by Meschi et al. [31]. This discrepancy may be attributed to the utilization of distinct autologous platelet concentrates (PRGF and A-PRF in the first three studies and L-PRF in the last one) and variations in the centrifugation and preparation methods employed, which could potentially influence the biological properties and molecular characteristics of the platelet concentrate. The proposed mechanisms underlying the alleviation of pain and reduction of inflammation induced by platelet products are linked to the local reduction of inflammatory factors, such as phospholipase A2 (PLA2), interleukin-1α (IL-1α), IL-1β, IL-6, IL-8, tumor necrosis factor-α (TNF-α), and prostaglandin E2 (PGE2). Furthermore, the release of growth factors and cytokines from platelet α granules, which have local anti-inflammatory, anti-apoptotic, and analgesic effects (e.g., via cytokines like IL-4 or IL-10), as well as their involvement in extracellular matrix production (ECM) and neural regeneration, are believed to be the primary mechanisms responsible for the benefits of APCs [40]. 

In general, while PRGF, A-PRF, and L-PRF have the potential to contribute to pain relief through their growth factors, anti-inflammatory properties, and tissue regeneration abilities, the differences in their composition and preparation methods can result in variations in their antinociceptive effects. The choice of platelet concentrate may depend on the specific clinical situation and the desired therapeutic outcome.

In addition, pain, swelling, and, in general, the extent of postoperative discomfort, are influenced by other several factors, including the complexity of the procedure, the time of surgery, the tissue trauma, the patient’s overall health, and the quality of postoperative care. In this regard, microsurgery in endodontics has revolutionized the field, offering precise and minimally invasive techniques that could be more tolerable for patients than the conventional ones and enhance the healing and success rates of complex endodontic procedures with respect to the past. Moreover, the use of piezosurgery reported by Ahmed et al. [25] during the procedure, may have an adjunctive role in reducing postoperative swelling and pain [41]; thus, the role of APCs in reducing pain, inflammation, and swelling may be partially masked if a microsurgical approach was used.

Otherwise, contradictory results were observed in this review about the effect of APCs on reduction in apical radiolucency. The osteogenic potential of autologous platelet concentrates has garnered significant attention in the field of regenerative medicine, particularly in oral and maxillofacial surgery, orthopedics, and implantology, especially in post-extraction socket healing [42,43], the osseointegration of dental implants, sinus lift procedures, and the healing of periodontal bone defects [44,45].

The osteogenic potential of these products has been widely demonstrated. Platelet concentrates contain growth factors like bone morphogenetic proteins (BMPs), Platelet-Derived Growth Factor (PDGF), and Transforming Growth Factor-beta (TGF-β), which can activate and accelerate the activity of osteoblasts. These factors play a vital role in bone regeneration and repair. Moreover, they improve the migration of various cells involved in bone regeneration, including osteoblasts, osteoclasts (cells responsible for bone resorption), and mesenchymal stem cells (MSCs), which are essential for the remodeling of bone tissue and the establishment of a well-vascularized bone matrix [46,47,48].

Nonetheless, the clinical effectiveness of APCs in bone regeneration procedures remains a subject of debate due to varying outcomes documented in various clinical applications [49,50,51,52], which is also substantiated by the results of this review. Bone healing is a complex and highly orchestrated biological phenomenon that involves a sequence of events that lead to the regeneration and restoration of damaged or lost bone tissue. In this review, the following several methods and tools in different period time are used to measure periapical bone healing: CBCT or periapical radiographs at 3 months [34], 9 months [32], or 1-year. 

Although periapical radiographs are one of the most used methods to evaluate bone healing, the CBCT has reported as a reliable method for monitoring reduced osseous lesion size and volume due to its three-dimensional measurement. Moreover, CBCT may be a valuable tool in conducting follow-ups in endodontics, even if the amount of ionizing radiation to which the patient is exposed is greater compared to single periapical radiographs [53,54]. In many cases, a combination of several assessment methods may be employed to provide a comprehensive evaluation of bone healing progress.

The influence of different follow-up intervals on bone healing can provide valuable insights into the healing process. In the first weeks of follow-up, radiographic imaging can reveal the development of callus formation, which is a key indicator of ongoing bone healing. In the long-term follow-up (months to a year). radiographic imaging assesses the quality and density of the healed matured bone. The different times of follow-up may influence the effectiveness of treatment. Moreover, in some cases, the use of biomaterials in adjunct to APCs may affect the reliability of radiographic evaluation [55]. The decision to use APCs alone or in combination with biomaterials is context-specific and depends on factors such as the type of tissue being treated, the size of the lesion, and the desired therapeutic goals. Since most included studies have demonstrated that graft materials showed no additional benefit when compared to APCs alone [25,34], a self-derived source of regenerative agents is advantageous in that it reduces the risk of immune rejection or adverse reactions. Moreover, where the body’s natural regenerative mechanisms are sufficient, like in four-wall-sided defects of apical lesions, APCs alone may be suitable. However, when more extensive tissue repair and regeneration are required, the strategic combination of APCs with biomaterials may probably offer a superior approach [56].

Indeed, contradictory results about soft and hard tissue healing between different APCs may be explained according to their composition and preparation methods. For example, PRF has been shown to be significantly better in promoting soft tissue healing and faster regeneration of bone after third molar extraction in comparison to PRP, and this could be attributed to simpler preparation protocols of PRF over PRP and the ability of PRF to release growth factors in a controlled way [57].

### 4.1. Risk-of-Bias Judgement of Eligible Studies

For RCTs, five studies had a low risk of overall bias, three moderate, and only one high. The domain that posed the greatest challenge was ensuring that patients remained unaware of their treatment allocation, and also whether those responsible for administering the treatment were similarly blinded to the treatment assignments. This difficulty arose not due to a methodological error but was a result of practical constraints since the venipuncture made for APCs preparation identified the intervention, and ethical reasons precluded the drawing of blood in both groups. Another risk to be highlighted is the blinding of outcome assessors when evaluating periapical radiography or CBCT, especially when a radiopaque bone graft serves as a control. In such instances, the presence of the bone graft may potentially obscure the assessment of bone healing and lead to an underestimation of lesion size reduction and bone density compared to the control group. 

### 4.2. Limitations 

Although the findings obtained from the present review suggest that the use of APCs during the endodontic surgical procedure is related to lower levels of pain, swelling, and swelling in the early post-surgical period, as well as to a reduction in apical radiolucency in the first 12 months of follow-up, it is necessary to reiterate its limitations. Substantial variation in clinical predictability and efficacy could be related to several variables, such as study design, defect type and location, type of surgical approach, APC preparation protocol, and patient response. In the included studies, the following different APCs prepared by different protocols were found: Angerame et al. [26] made use of PRF (2500 rpm for 10 min), Del Fabbro M et al. [27] instead made use of PRGF (3200 rpm for 8 min), Goyal et al. [29] still used PRP (2400 rpm for 10 min followed by 3600 rpm for 15 min) while Soto-Penaloza [35] made use of A-PRF (1300 rpm for 8 min). This did not allow for a uniform and homogeneous evaluation of the data, even though all APCs individually demonstrated improved clinical outcomes. This heterogeneity was also found in the surgical procedure used in terms of the type of surgical access, execution of the bone breach, cutting of the root apex, preparation of the apical cavity, and material used for retrograde root filling. Unfortunately, the high heterogeneity of the studies in terms of APCs and surgical protocol used does not allow for drawing definitive conclusions and fails to provide valid clinical guidelines for the use of APCs in surgical endodontics. 

### 4.3. Prospective 

Employing regenerative techniques with APCs in the clinical practice of endodontic surgery holds the potential to enhance the recovery of periapical lesions. In the realm of clinical research, forthcoming trials should carefully consider the influence of lesion type and size on the effectiveness of these products. However, it is recommended not to use bone graft materials in combination with APCs, as they did not show any significant difference and offered only extra financial cost to the patient. Additionally, researchers may explore different combinations of APCs to optimize their impact on wound healing after endodontic surgery and conduct studies with more homogeneous techniques and outcome measurements that can be compared and subjected to meta-analysis.

## 5. Conclusions

Within its limits, the present systematic review showed the effectiveness of APCs in reducing the pain and swelling in the early post-surgical period, as well as an improvement in daily activities and quality of life in patients undergoing endodontic surgery. However, the high heterogeneity of the studies in terms of APCs and surgical protocol used does not allow for drawing definitive conclusions. Additional research with expanded sample sizes, extended follow-up periods, and standardized protocols is necessary to gain a more comprehensive understanding of the role of autologous platelet concentrates in endodontic surgery.

## Figures and Tables

**Table 1:** Search strategy.

Pubmed	(“endodontics” [MeSH Terms] OR “endodontic*” [All Fields] OR “endodontic surgery” [All Fields] OR “root-end surgery” [All Fields] OR “root-end resection” [All Fields] OR “surgical endodontic treatment” [All Fields] OR “endodontic healing” [All Fields] OR “apicoectomy” [MeSH Terms] OR “periapical surgery” [All Fields] OR “endodontic microsurgery” [All Fields]) AND (“platelet concentrates” [All Fields] OR “autologous platelet concentrates” [All Fields] OR “platelet-rich plasma” [All Fields] OR “Platelet-rich fibrin” [All Fields] OR “hemocomponents” [All Fields] OR “platelet rich in growth factors” [All Fields] OR “PRGF” [All Fields] OR “PRP” [All Fields] OR “PRF” [All Fields])
Scopus	(TITLE-ABS-KEY (endodontic*) OR TITLE-ABS-KEY (“endodontic surgery”) OR TITLE-ABS-KEY (“root-end surgery”) OR TITLE-ABS-KEY (“root-end resection”) OR TITLE-ABS-KEY (“surgical endodontic treatment”) OR TITLE-ABS-KEY (“endodontic healing”) OR TITLE-ABS-KEY (apicoectomy*) OR TITLE-ABS-KEY (“periapical surgery”) OR TITLE-ABS-KEY (“endodontic microsurgery”)) AND (TITLE-ABS-KEY (“platelet concentrates”) OR TITLE-ABS-KEY (“autologous platelet concentrates”) OR TITLE-ABS-KEY (“platelet-rich plasma”) OR TITLE-ABS-KEY (“Platelet-rich fibrin”) OR TITLE-ABS-KEY (hemocomponents))
Cochrane Oral Health Group Databases	(platelet concentrates in endodontic surgery);ti,ab,kw

**Table 2:** Study characteristics: CCT, controlled clinical trial; RCT, randomized clinical trial; PRF, platelet-rich fibrin; BG, bioactive glass; PRGF, plasma rich in growth factor.

Author, Year of Publication	Country	Type of Study	Total. n. of Patients, Age; Gender	Diagnosis	Intervention	Control	Follow-Up	Diagnostic Method	Outcome	Clinical Results	Radiographical Results
Ahmed G.M. et al., 2018 [25]	Egypt, Cairo	RCT	12 patients, from 18 to 45 years old; 9 female and 3 males	Periapical lesions in maxillary anterior teeth	Endodontic microsurgery with root-end preparations by ultrasonic retrotips and by filling with PRF gel or combined PRF gel and bioactive glass		1 year follow-up	Cone beam computed tomography (CBCT)	Bone healing after endodontic microsurgery using PRF and PRF-BG		Decrease in the periapical lesion volume and healing of the periapical defects
Angerame D. et al., 2015 [26]	Italy, Trieste	RCT	11 patients aged from 28 to 72: 6 female and 5 males	Chronic apical periodontitis	Endodontic microsurgery with root-end resection and retrograde filling	In the control group the bone defect was not filled	1 year follow-up	Digital X-ray system was used for radiographic examination. A questionnaire was adopted for pain and swelling information.	Radiographic healing and the postoperative discomfort in patients undergoing apical surgery, either by leaving the apical surgical cavity empty or by filling it with the PRF gel	PRF test group felt less intense pain and swelling than the control group during the first hours and days postoperatively	PRF test group exhibited significantly better periapical healing scores than the control group
Del Fabbro M. et al., 2012 [27]	Italy, Milan	RCT	36 patients; aged from 34 to 62; 20 women and 16 men	Chronic apical periodontitis	Endodontic microsurgery with retrograde root canal preparation: in the test group a thin layer of liquid PRGF was applied into the root-end and the defects was filled with a clot of PRGF	The control group was treated with modern endodontic surgery alone without using PRGF	7 postoperative days	A quality of life questionnaire was adopted to evaluate postoperative limitations in function as well as pain and the presence of other symptoms.	Evaluate whether the use of PRGF might have a favorable impact on patient’s quality of life after endodontic surgery.	The test group showed significantly less pain and swelling, fewer analgesics taken, and improved functional activities as compared with the control group.	
Dhiman M. et al., 2015 [28]	India, Haryana	RCT	30 patients aged 17–47: 11 women and 19 men	Suppurative chronic apical periodontitis and apicomarginal communication	Endodontic surgery with root-end resection, preparation, and root-end filling. In the test group a PRF membrane was been placed into the bone defect	In the control group no PRF membrane was used	1 year follow-up	Follow-up radiographs were compared with postoperative radiographs	Healing of apicomarginal	PD showed a statistically significant reduction in the PRF group	Reduction of apical radiolucency in the test group after the 12 months follow-up
Goyal B. et al., 2011 [29]	India, Haryana	CCT	30 patients divided into 3 groups; aged 17–45; 13 women and 17 men	Suppurative chronic apical periodontitis and apicomarginal communication	Endodontic surgery with root-end resection, preparation, and root-end filling. Subjects were assigned to the collagen membrane group, PRP group, and PRP + collagen sponge group without stratification.	In the control group collagen membrane (GTR) was shaped and placed over the defects	1 year follow-up	The clinical parameters were measured on the buccal aspect of the interproximal space and the midbuccal aspect of the involved teeth using a periodontal Williams O probe. The radiographs were taken with the Rinn parallel technique	Compare the healing responses of platelet-rich plasma (PRP), PRP + a collagen sponge, and a collagen membrane	All the three treatments showed highly significant reductions in the PD, CAL, and in gingival margin position	All the three treatments showed highly significant radiographic reduction of the size of the periapical lesion, the percentage reduction of the periapical rarefactions and periapical healing
Meschi N. et al., 2018 [30]	Belgium, Leuven	RCT	50 patients divided into two groups; aged 16–79; 28 women and 22 men	Patients in need of root-end surgery due to periapical lesions	Endodontic microsurgery with root-end resection, preparation, and filling. In the test group, LPRF with or without BG was placed before flap repositioning.	In the control group was not added LPRF	1 week post RES	Patients completed daily for 1 week a visual analog pain scale (VAS) and a 5-point Likert-type scale questionnaire	Impact of the adjunct of leukocyte- and platelet-rich fibrin (LPRF) to root-end surgery (RES) on the patients’ quality of life during the first week post RES.	No evidence for a difference between the test and control group in VAS, occurrence of pain symptoms, impairment of daily activities, and medication use, over the 7 days and daily during the 7 days post RES.	
Meschi N. et al., 2020 [31]	Belgium, Leuven	RCT	50 patients divided into four groups; aged 27–57; 28 female and 22 men	Patients in need of root-end surgery due to periapical lesions	3 mm root-end resection, and removal of the cyst or granuloma; root-end preparation of 3–5 mm with an ultrasonic device and root-end filling with mineral trioxide aggregate	Application of a BG membrane, or nothing	1 year post RES	Ultrasound imaging, periapical radiographs, and cone-beam computed tomography were used to evaluate bone healing	Periapical bone healing		The addition of an occlusive membrane rather than an autologous platelet concentrates improved bone regeneration 1 year post RES significantly, irrespective of the assessment device applied
Monga P. et al., 2016 [32]	India, Punjab	CCT	30 patients divided into 3 groups (A, B and C)	Periapical lesions such as granulomas and cysts	Endodontic surgery with root-end resection, preparation, and root-end filling. In Group A, root-end cavity was filled with MTA. In Group B, with MTA followed by placement of hydroxyapatite in the curetted periapical defect. In Group C, with MTA followed by placement of PRF in the curetted periapical defect.		9 months follow-up	Patients were examined clinically regarding postoperative discomfort, pain, sensitivity to percussion, and presence/absence of swelling. Radiographically, an intraoral periapical radiograph as taken on each follow-up visit.	Effectiveness of periapical surgery using MTA as a retrograde filling material with/without using hydroxyapatite or PRF in curetted periapical defect		A significantly higher rate of healing was observed after 9 months when apicoectomy was performed using retrograde filling materials with PRF as a graft material in Group C followed by hydroxyapatite n Group B as compared to Group A, where no graft material was added in the curetted periapical defect
Parihk B. et al., 2011 [33]	India, Baroda	CCT	24-year-old male patient	Upper central incisors fractured and bilateral periapical radiolucency	RES with cystic enucleation bilaterally; retrograde cavity preparations were performed along the long axis of the tooth to a depth of 3–4 mm; MTA was used as a root-end filling material	PRP was applied in only one unilateral incisor	2 years follow-up	Vitality test, radiographic endoral and spiral computed tomography	Healing periapical lesions treated with and without the use of platelet- rich plasma		Definite healing seen in the lesion treated with PRP on the radiograph
Singh R. et al., 2020 [34]	India, Rajasthan	CCT	126 patients aged between 18 and 38 years divided into 3 groups	Periapical lesions such as periapical abscess, cyst, and granuloma in the maxillary anterior region	Endodontic surgery with root-end resection, preparation, and root-end filling.	In the other two groups CERAMENT and hydroxyapatite were used	1 year follow-up	Preoperative and postoperative radiographs were taken by following the paralleling technique. Vitality was evaluated with thermal testing.	Compare hydroxyapatite granules, CERAMENT, and platelet-rich fibrin (PRF) in the management of endodontic apical surgery cases	PRF is superior in terms of reducing pain, mobility, and sinus as compared to hydroxyapatite and CERAMENT	Definite healing seen in the lesion treated with A-PRF on the radiograph
Soto-Penaloza D. et al., 2020 [35]	Spain, Valencia	RCT	50 patients divided into 2 groups; aged 17–79; 28 women and 22 men	50 apical lesions of upper maxilla (second premolar to the second premolar)	Endodontic surgery with root-end resection, preparation, and root-end filling. In the test group A-PRF + membranes were placed inside the bony crypt	In the control group was not added A-PRF	1 week after surgery	Pain perception and quality of life (functional limitations and other symptoms) were assessed 1 week after surgery using a visual analog scale and a Likert scale-based questionnaire.	Postoperative pain, and quality of life in endodontic	Pain perception was mild in both groups versus; it proved less variable during the first 4 days in test group, showing lower extreme pain values.	
Taschieri S. et al., 2013 [36]	Italy, Milan	CCT	20 patients divided into 2 groups; aged 29–55; 11 female and 9 males	Patients treated by microsurgical endodontic treatment in molar and premolar maxillary region.	Endodontic retrograde treatment	In the control group platelet concentrates was not used	1 year follow-up	A questionnaire was administered to all subjects to evaluate postoperative functional limitations as well as pain and the presence of other symptoms. Periapical radiographs were taken at follow-up	Postoperative quality of life	Significantly improved patients’ quality of life was observed in the test group considering symptoms as swelling, or taste, and pain. Also, functional activities were less impaired in the test group.	
Thakur V. et al., 2023 [37]	Haryana, India	RCT	40 patients divided into the following 2 groups: test group with PRF High and control group with PRF Medium (20 male, 20 female).	Patients presenting with endodontic lesions and concomitant periodontal communication.	Endodontic surgery with root-end resection, preparation, and root-end filling.	In the control group PRF-Medium was used.	1 week after surgery for clinical parameters and 1 year follow-up for radiographical parameters	Quality of life questionnaire and visual analogic scale (VAS) for clinical outcomes and CBCT for radiographic outcomes.	Postoperative quality of life and periapical bone healing.	PRF Medium group patients re- ported significantly less swelling on the 1st and 3rd days, and average pain on the 2nd and 4th days postoperatively.	The difference in success rate for periapical healing was non-significant between the PRF Medium group and PRF High group in both 2D and 3D imaging.
Yahata Y. et al., 2023 [38]	Japan	RCT	24 patients (8 male, 16 female) divided into two groups.	Patients in need of root-end microsurgery due to periapical lesions.	Root-end resection was accomplished approximately 3 mm from the apex. The root-end cavity preparation was performed using ultrasonic microtips up to a depth of 3 mm in the canal space along the long axis.	In the control group no CGF was used.	1 year follow-up with follow-up cone-beam computed tomography (CBCT) at 6 months.	Periapical radiography and CBCT for radiographic parameters and VAS score for clinical parameters.	Postoperative discomfort and periapical bone healing.	Regard to the VAS scores, there was no significant difference between the two groups preoperatively and at all postoperative appointments.	The lesion volume reduction rate in the CGF group (75.6%) was significantly higher than that in the control (61.0%) group.

PD, probing depth; PRP, platelet-rich plasma; GTR, guided tissue regeneration; CAL, clinical attachment loss; L-PRF, leucocyte-platelet rich fibrin; RES, root-end surgery; VAS, visual analogue scale. MTA, mineral trioxide aggregate.

**Table 3:** Assessment of quality and risk of bias for randomized controlled trials (RCT) included in the systematic review. Each domain was satisfied (yes), not satisfied (no), unclear, or not assessable (N/A) according to the Joanna Briggs Institute Critical Appraisal tool.

Study	Was True Randomization Used for Assignment of Participants to Treatment Groups?	Was Allocation to Treatment Groups Concealed?	Were Treatment Groups Similar at the Baseline?	Were Participants Blind to Treatment Assignment?	Were Those Delivering Treatment Blind to Treatment Assignment?	Were Outcomes Assessors Blind to Treatment Assignment?	Were Treatment Groups Treated Identically Other than the Intervention of Interest?	Was Follow-Up Complete and if Not, Were Differences between Groups In Terms of Their Follow-Up Adequately Described and Analyzed?	Were Participants Analyzed in the Groups to Which They Were Randomized?	Were Outcomes Measured in the Same Way for Treatment Groups?	Were Outcomes Measured in a Reliable Way?	Was Appropriate Statistical Analysis Used?	Was the Trial Design Appropriate, and Any Deviations from the Standard RCT Design in the Conduct and Analysis of the Trial?	Overall Risk of Bias
Angerame D. et al., 2015 [26]	YES	YES	YES	YES	UNCLEAR	UNCLEAR	YES	NO	UNCLEAR	YES	YES	YES	YES	moderate
Ahmed G.M. et al., 2018 [25]	YES	YES	YES	UNCLEAR	UNCLEAR	YES	YES	YES	YES	YES	YES	YES	YES	low
Del Fabbro M. et al., 2012 [27]	YES	YES	YES	UNCLEAR	UNCLEAR	YES	YES	YES	YES	YES	YES	YES	YES	low
Dhiman M. et al., 2015 [28]	YES	YES	YES	UNCLEAR	UNCLEAR	YES	YES	YES	YES	YES	YES	YES	YES	low
Meschi N. et al., 2018 [30]	YES	YES	YES	UNCLEAR	UNCLEAR	YES	YES	YES	YES	YES	YES	YES	UNCLEAR	moderate
Meschi N. et al., 2020 [31]	YES	YES	YES	UNCLEAR	UNCLEAR	YES	YES	YES	YES	YES	YES	YES	UNCLEAR	moderate
Soto-Penaloza D. et al., 2020 [35]	YES	YES	YES	UNCLEAR	UNCLEAR	YES	YES	YES	YES	YES	YES	YES	YES	low
Thakur V. et al., 2023 [37]	YES	YES	YES	YES	YES	UNCLEAR	YES	YES	UNCLEAR	YES	YES	YES	YES	low
Yahata Y. et al., 2023 [38]	YES	YES	YES	YES	YES	UNCLEAR	YES	YES	UNCLEAR	YES	YES	YES	UNCLEAR	low

**Table 4:** Assessment of quality and risk of bias for quasi-experimental studies included in the systematic review. Each domain was satisfied (yes), not satisfied (no), unclear, or not applicable (N/A) according to the Joanna Briggs Institute Critical Appraisal tool.

Study	Is It Clear in the Study What Is the ‘Cause’ and What Is the ‘Effect’?	Were the Participants Included in Any Comparisons Similar?	Were the Participants Included in Any Comparisons Receiving Similar Treatment/Care, Other than the Exposure or Intervention of Interest?	Was There a Control Group?	Were There Multiple Measurements of the Outcome Both Pre and Post the Intervention/Exposure?	Was Follow-Up Complete and if Not, Were Differences between Groups in Terms of Their Follow-Up Adequately Described and Analyzed?	Were the Outcomes of Participants Included in Any Comparisons Measured in the Same Way?	Were Outcomes Measured in a Reliable Way?	Were Appropriate Statistical Analysis Used?	Overall Risk of Bias
Goyal B. et al., 2011 [29]	YES	YES	YES	YES	NO	NO	YES	YES	YES	moderate
Monga P. et al., 2016 [32]	YES	YES	YES	YES	NO	NO	YES	YES	YES	moderate
Parihk B. et al., 2011 [33]	YES	NOT APPLICABLE	NOT APPLICABLE	YES	NO	UNCLEAR	YES	UNCLEAR	YES	high
Singh R. et al., 2020 [34]	YES	YES	YES	YES	NO	YES	YES	UNCLEAR	YES	moderate
Taschieri S. et al., 2013 [36]	YES	YES	YES	YES	YES	YES	YES	YES	YES	low

## Data Availability

No new data were created or analyzed in this study. Data sharing is not applicable to this article.
